# Harnessing Generative Artificial Intelligence to Teach Assessment Literacy in Anatomy

**DOI:** 10.1007/s40670-026-02659-x

**Published:** 2026-02-07

**Authors:** Alicia Morgan, Marli Crabtree, Caitlin Sachsenmeier, Amanda Troy

**Affiliations:** Department of Anatomy, Duquesne University Nasuti College of Osteopathic Medicine, Pittsburgh, PA USA

**Keywords:** Artificial intelligence, Anatomy, Medical education, Item writing, Assessment, Critical appraisal

## Abstract

Incoming medical students attended an anatomy bootcamp introducing prompt engineering and critical appraisal of AI-generated questions. MBS students completed an AI-assisted item-writing assignment requiring generation, appraisal, and revision that was used to create their unit review session. Both experiences nurtured ownership, assessment literacy, and critical reasoning while reinforcing anatomy learning.

Generative artificial intelligence (GAI) is increasingly promoted for its ability to create multiple-choice questions (MCQs), and many medical students now use it as part of their preparation for assessments and licensure examinations [1]. Yet, students are rarely trained in how to engage with these tools responsibly or critically [2]. At Duquesne University Nasuti College of Osteopathic Medicine, we sought to address this gap during our inaugural anatomy bootcamp for incoming osteopathic medical students (OMS1) and Master of Biomedical Sciences (MBS) students during their anatomy coursework.

The three-day bootcamp was designed to help incoming medical students—many of whom had never studied anatomy—gain confidence with the language of the subject and practice evidence-based study strategies. Alongside hands-on, team-based retrieval activities (*n* = 10), we delivered a three-part lecture series (one per day) on “Studying in Medical School,” outlined in Table 1, which included one part focused on GAI. Some students also experimented with a custom Generative Pre-Trained Transformer (GPT) created by one of the instructors; this GPT-based resource generates MCQs aligned with course learning objectives (LOs), provides SWOT analyses post-assessment, and creates adaptive assessments that build on students’ past performance. Students valued the opportunity to explore this tool in a structured and supervised environment, rather than relying on AI informally and without guidance. In light of GAI’s environmental footprint [3] and the time invested by our already busy students in carefully appraising and rekeying questions, students are encouraged to share their completed items as a collaborative study resource for course preparation and board exams.

**Table 1:** Overview of the “Studying in medical School” anatomy boot camp and MBS item-writing interventions with respective cohort feedback

Participants	DO Anatomy Boot Camp	MBS Item Writing
	54 OMS1	60 MBS students
Activity	*3-part lecture series (1 h/session)*: • **Part 1**: metacognitive study strategies for anatomy • **Part 2**: MCQ deconstruction and rekeying • **Part 3**: effective use and critical appraisal of GAI for question practice and tracking progress in LOs	1. Select a lecture LO from the unit. 2. Create a COMLEX-style MCQ using either own knowledge or chosen GAI (with clear disclosure statement). 3. Provide 5 answer choices with 1 best answer and explanations for each. 4. Critically appraise the item using a rubric created by course instructors. 5. Revise the question based on appraisal. 6. Tag item with 3 key words to encourage organization for later use.
Primary feedback themes (*voluntary surveys*)	**New ways to use AI** • as a tool for studying • future uses in medicine **Useful discussion** • become aware of uses/issues • should understand how it’s used **Improvement of study techniques** • new level of learning with practice questions • creation of custom GPT **Acknowledging flaws of AI** • inaccuracies	**Assessment literacy** • role of distractors • wording can influence difficulty • how questions assess application vs. recall **Metacognitive benefits** • reflect on knowledge/LO gaps • engage more in practice questions independently **AI as a scaffold** • hesitant about accuracy without faculty oversight • useful in generating alternative answers or checking clarity

For our MBS students, a one-day program orientation included a brief introduction to the anatomy of a question and general study tips, but had no focus on GAI or building practice questions. However, MBS students encountered intentional AI-based item writing assignments during their anatomy courses. These assignments are intended to promote higher-order thinking, provide insight on using assessment-aligned practice questions, and support the transition toward a medical student learning mindset. Table 1 details the first item writing assignment for MBS students. While students could generate their first draft with AI, the central expectation was critical appraisal and revision. In an appraisal rubric, students reflected on topics like clarity and focus of the question, difficulty level, anatomical accuracy, and plausibility of distractors. Nearly all students chose to use GAI, most commonly ChatGPT. Course instructors then evaluated and curated all completed assignments into a 60-question review session that students used in preparation for the written exam with minimal supplementation for any missed LOs.

For the spring course, instructors adapted this assignment to provide another way for MBS students to generate their own practice questions. Students designed an initial question, then revised with intention of re-keying, altering the stem and/or distractors so that one of the original incorrect options becomes the correct choice. The re-keying process required students to critically re-evaluate the anatomical relationships underlying each distractor, analyze why each was originally incorrect, and intentionally modify the question stem to align with a new correct answer.

Overall, OMS1 and MBS student responses between the different experiences have been enthusiastic, with response themes detailed for each cohort in Table 1. Out of 10 boot camp activities, medical students ranked all parts of the “Studying in Medical school” series within the top 4, with part 3 (prompt engineering/GAI in MCQ generation) as 2/10. Learners noted that while many had already been experimenting with GAI, the structured environment gave them a safe space to practice, receive guidance, and learn boundaries for responsible use. They reported that the opportunity to critique and revise questions strengthened both their understanding of anatomy and construction of higher-quality assessment items for their own practice. In general, MBS students seemed more hesitant on AI use, preferring instructor-made questions. Several MBS students reported that manual item writing promoted deeper learning but required more time than they were willing to allocate to a single assignment.

Our early experiences suggest that GAI can serve not only as a tool for generating practice questions but also as a pedagogical partner in teaching assessment literacy when incorporated with intention. Providing structured opportunities for students to engage with GAI—anchored in LOs, item-writing principles, and faculty oversight—may help mitigate risks of overreliance while enhancing preparation for high-stakes assessments. By shifting the emphasis from consuming AI-generated questions to appraising and revising them, we empower students to become co-authors in the design of their own learning and assessment.

## Declarations

The authors used OpenAI, GPT- 5 (accessed September 2025) to assist with minor language editing. The authors reviewed and are responsible for the final content.
